# The epigenetic origin of life history transitions in plants and algae

**DOI:** 10.1007/s00497-021-00422-3

**Published:** 2021-07-08

**Authors:** Jérômine Vigneau, Michael Borg

**Affiliations:** Department of Algal Development and Evolution, Max Planck Institute for Developmental Biology, Tübingen, Germany

## Abstract

Plants and algae have a complex life history that transitions between distinct life forms called the sporophyte and the gametophyte. This phenomenon—called the alternation of generations—has fascinated botanists and phycologists for over 170 years. Despite the mesmerizing array of life histories described in plants and algae, we are only now beginning to learn about the molecular mechanisms controlling them and how they evolved. Epigenetic silencing plays an essential role in regulating gene expression during multicellular development in eukaryotes, raising questions about its impact on the life history strategy of plants and algae. Here, we trace the origin and function of epigenetic mechanisms across the plant kingdom, from unicellular green algae through to angiosperms, and attempt to reconstruct the evolutionary steps that influenced life history transitions during plant evolution. Central to this evolutionary scenario is the adaption of epigenetic silencing from a mechanism of genome defense to the repression and control of alternating generations. We extend our discussion beyond the green lineage and highlight the peculiar case of the brown algae. Unlike their unicellular diatom relatives, brown algae lack epigenetic silencing pathways common to animals and plants yet display complex life histories, hinting at the emergence of novel life history controls during stramenopile evolution.

## The alternation of generations

**Fig. 1:**
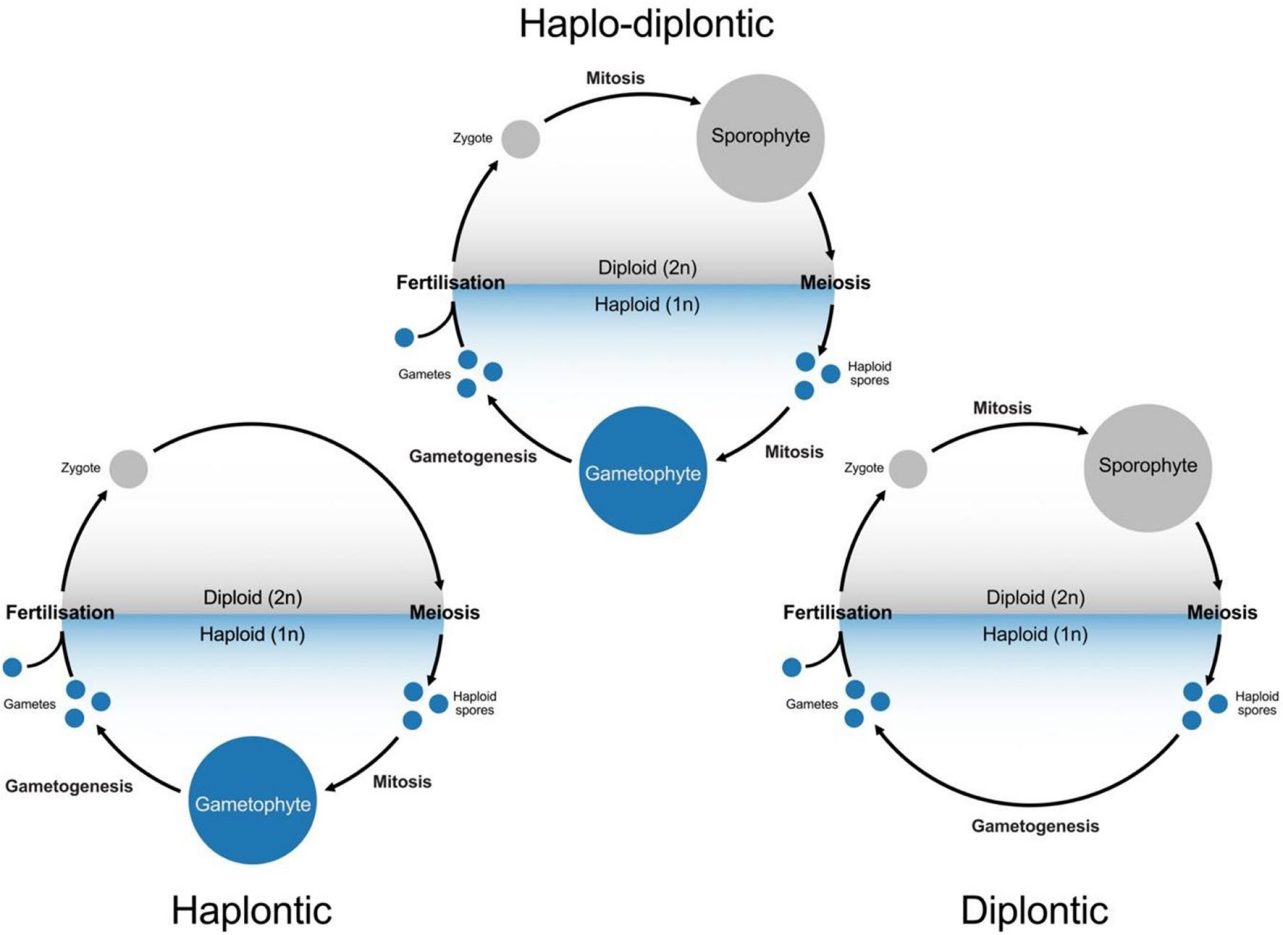
Life cycle schemes in living organisms. Haplo-diplontic life cycles are characterized by mitotic divisions in both the diploid zygote and haploid meiotic spores, which produce distinct and sometimes free-living sporophytes and gametophytes, respectively. The diploid zygote immediately undergoes meiosis in haplontic life cycles, where mitotic divisions of the resulting haploid spores generate a large multicellular individual or more unicellular haploid cells. Conversely, the diploid zygote in organisms with a diplontic life cycle divides mitotically to produce a multicellular individual or more unicellular diploid cells. Once diploid cells of diplontic organisms undergo meiosis, the resulting haploid cells directly differentiate into the gametes

The alternation of generations is a developmental phenomenon where two distinct life forms—the sporophyte and gametophyte—alternate in the life cycle of plants and algae. The sporophyte is diploid and gives rise to haploid spores at meiosis that go on to develop into gamete-producing gametophytes. The union of gametes at fertilization initiates the formation of the diploid sporophyte to complete this so-called *haplo-diplontic* life cycle (Fig. 1). The classical studies of Wilhelm Hofmeister were the first to establish the universal occurrence of two life forms in plants (Hofmeister 1851, 1862). Hofmeister keenly recognized the presence of two separate, free-living generations in the land plant life cycle, despite substantial varying morphologies among the major plant groups. What ensued was a debate among scientists of the late nineteenth century about the origin of these alternating generations, and the key discovery that these alternations also included changes in chromosome number or ploidy. The history and outcome of these classical debates can be delved into by the reader in excellent in-depth reviews on the subject (Bell 1989; Haig 2008).

An obvious advantage of a complex life cycle is the adaptation of life forms to distinct functional roles (Dickinson and Grant-Downton 2009). The sporophyte and gametophyte often exhibit substantial differences in physical size, cell types and duration spent in either phase (Bell 1989). A striking evolutionary trend observed in the plant and brown algal lineages, both of which evolved complex multicellularity, is a tendency for a dominant diploid phase and a concomitant reduction in the number of mitotic divisions and cell types of the haploid phase (Rudall 2006a; Rudall and Bateman 2007a). The life cycle of angiosperms serves as an apt example, where the diploid sporophyte represents the bulk of the vegetative plant body while the inconspicuous pollen grain and ovule represent the male and female gametophytes, respectively. A perspective on the adaptive qualities of alternating life cycles and the emergence of the sporophyte as the dominant phase can be garnered elsewhere (Graham and Wilcox 2000; Haig and Wilczek 2006).

As a developmental process, the alternation of generations is remarkable in that a single genome can express multiple morphologically distinct life forms. While fertilization and meiosis represent the transition checkpoint between these life forms (Fig. 1), changes in ploidy level alone do not appear to be sufficient to initiate these developmental transitions. Haploid sporophytes are readily produced through in vitro anther culture in several angiosperms (Hu and Guo 1999; Lv et al. 2020; Wang et al. 2021), while diploid gametophytes are observed in some developmental mutants in the brown algae (Coelho et al. 2011a; Arun et al. 2019). Moreover, the gametes in several algal species can develop into haploid sporophytes through parthenogenesis and thereby bypass fertilization altogether (Coelho et al. 2011b). How the marked differences in gene expression, development and morphology are established between the gametophyte and sporophyte is poorly understood and thus remains an important question in developmental biology.

Patterns of DNA and histone methylation, which control genomic activity through transcriptional repression, are reprogrammed multiple times during germline differentiation and early embryogenesis in mammals (Morgan et al. 2005). This erasure of epigenetic marks is called epigenetic reprogramming and it serves to reshape the transcriptional landscapes that specify the gametes and development of the embryo (Feng et al. 2010). It thus stands to reason that the epigenetic patterns established in the gametophyte or sporophyte undergo reprogramming during the alternation of generations. There have been extensive cellular and molecular investigations of the sporophyte and gametophyte, particularly in plants, but these tend to occur in isolation and often without consideration of the alternation of generations (Dickinson and Grant-Downton 2009). At the level of epigenetics and genomics, less progress has been made due to the difficulty of isolating the microscopic cells of the less dominant generation. Here, we undertake a comprehensive review of epigenetic studies in light of the evolution and control of the alternation of generations. We critically assess the existing literature and highlight more recent studies that have directly addressed this phenomenon in *Arabidopsis*, then postulate how epigenetic silencing might have been adapted to regulate life history transitions during plant evolution. We end by discussing the phylogenetically distinct stramenopile lineage and highlight the independent evolutionary origin of the alternation of generations in brown algae.

## Epigenetic modifications are few and far between

There is much confusion in the literature over the term ‘epigenetic’ since it is often used to encompass all forms of DNA and histone modification (Bird 2007). The most accurate and accepted definition of epigenetics defines it as ‘the study of heritable phenotypes that do not alter the DNA sequence’ (Bonasio et al. 2010; Arimondo et al. 2019). Just as genetic information encoded by DNA is inherited from cell-to-cell, so too must an epigenetic modification be faithfully restored during each cell cycle (Reinberg and Vales 2018). Bearing this in mind, only a handful of DNA and histone modifications should be considered truly epigenetic. With this in mind, we will briefly introduce the most *bona fide* examples of epigenetic marks that will form the basis for our ensuing discussion.

5′-methylcytosine (5mC)—the methylated form of the nucleotide cytosine—is the most widely studied epigenetic modification (Holliday and Pugh 1975; Bird 2002). In animals, 5mC occurs at CG dinucleotides, while in plants it can occur in CG, CHG and CHH dinucleotide contexts (where H denotes A, C or T) (Kawashima and Berger 2014). Methylation in each context plays distinct biological functions and is deposited by dedicated families of DNA methyltransferases (Law and Jacobsen 2010; Stroud et al. 2014). DNA methylation is largely confined to promoters and heterochromatic regions, where it mediates gene regulation, transposable element (TE) silencing and genome stability (Zhang et al. 2018). In addition, CG methylation is also enriched over the gene body of expressed genes (Bewick and Schmitz 2017). The symmetric nature of CG methylation results in its semi-conservative segregation during DNA replication, such that it is faithfully restored on newly synthesized strands of DNA through a template-copying mechanism (Zhu and Reinberg 2011). In animals, CG methylation is re-established by DNA methyltransferase 1 (DNMT1) through its recruitment to the replication fork by PCNA and UHRF1 where it preferentially methylates hemi-methylated DNA (Bostick et al. 2007).

In plants, VIM proteins represent the orthologs of mammalian UHRF1 that probably also act to recruit MET1, the plant ortholog of mammalian DNMT1 that restores CG methylation (Woo et al. 2008), while symmetric CHG methylation is restored by Chromomethylase 3 (CMT3) (Lindroth et al. 2001). In contrast, asymmetric CHH methylation is deposited by Domains Rearranged Methyltransferase 2 (DRM2) through RNA-directed DNA methylation (RdDM) and by CMT2 (Matzke et al. 2009; Zemach et al. 2013). A feedback loop mechanism is required where CMT2 and CMT3 recognize H3K9me2 to methylate neighboring CHH and CHG sites while the H3K9 methyltransferase KRYPTONITE (KYP) recognizes methylated cytosines, further reinforcing the transcriptional silencing of heterochromatin (Wilkins and Holliday 2009; Du et al. 2014; Niklas et al. 2014; Lenormand et al. 2016). In the yeast *Cryptococcus neoformans*, CG methylation is propagated by the maintenance methyltransferase DNMT5 in the absence of a de novo methyltransferase and also involves an H3K9 methylation loop (Catania et al. 2020). Symmetric CG and CHG methylation thus represent mitotically heritable epigenetic marks in plants, whereas non-symmetric CHH methylation requires de novo establishment during each cell cycle.

Chromatin disassembly during DNA replication results in the recycling of parental nucleosomes onto nascent strands of chromatin (Petryk et al. 2018; Reverón-Gómez et al. 2018; Schlissel and Rine 2019). This re-partitioning of modified nucleosomes provides a means for the epigenetic inheritance and restoration of histone modifications from mother-to-daughter cells (Petryk et al. 2018; Reverón-Gómez et al. 2018; Schlissel and Rine 2019). Euchromatic histone modifications like H3K9ac and H3K27ac as well as H3K4me1, H3K4me3 and H3K36me3, all of which play crucial roles in transcription, do not undergo maintenance across the cell cycle (Henikoff and Shilatifard 2011; Pérez-Lluch et al. 2015). Only two types of histone modification—the methylated forms of H3K9 and H3K27—have been demonstrated to be transmissible through cell division (Zhu and Reinberg 2011). The mitotic heritability of H3K9 methylation has been demonstrated in fission yeast (Grewal and Klar 1996; Nakayama et al. 2001; Grewal and Jia 2007; Audergon et al. 2015; Torres-Garcia et al. 2020), which involves a self-sustaining ‘read-write’ mechanism that is mediated by the recruitment of H3K9 methyltransferases to the replication fork (Reese et al. 2003; Sarraf and Stancheva 2004; Estève et al. 2006; Loyola et al. 2009; Li et al. 2011). In plants, H3K9me2 represents the equivalent of H3K9me3 and is catalyzed by KRYPTONITE (KYP)/SU(VAR)3-9 HOMOLOG 4 (SUVH4), SUVH5 and SUVH6 (Jackson et al. 2002; Du et al. 2012, 2014, 2015). Although likely to be conserved, direct evidence for the recruitment of these H3K9 methyltransferases to the replication fork in plants, which would mediate epigenetic inheritance of H3K9me2, has yet to be demonstrated.

A similar read–write mechanism exists to propagate H3K27 methylation across cell divisions. H3K27me3 is deposited by the widely conserved Polycomb Repressive Complex 2 (PRC2) (Hennig and Derkacheva 2009). PRC2 was first discovered from genetic screens in *Drosophila melanogaster* as a key regulator of homeotic genes during development (Schwartz and Pirrotta 2007). PRC2 is comprised of four proteins, with H3K27me3 deposition carried out by homologs of *Drosophila* SET domain methyltransferase Enhancer of Zeste (E(z)) (Schwartz and Pirrotta 2007). PRC2-mediated silencing of developmental genes is highly conserved across animals and plants and is essential for maintaining cellular identity during growth and development (Margueron and Reinberg 2011). In animals, PRC2 localizes to replication foci throughout S-phase where it propagates H3K27me3 domains across the cell cycle using pre-existing H3K27me3-marked nucleosomes as a template (Hansen et al. 2008; Margueron et al. 2009; Coleman and Struhl 2017; Laprell et al. 2017).

Similarly, the loss of PRC2 from chromatin dilutes H3K27me3 in a cell cycle–dependent manner during plant cell fate determination (Sun et al. 2014). PRC2 subunits have been shown to interact with components of the replication fork in *Arabidopsis*, which include the E(z) ortholog CURLY LEAF (CLF) (Jiang and Berger 2017). H3K27me3 maintenance involves the initial K27 mono-methylation of replicative histone H3.1 by the plant-specific SET domain methyltransferases ARABIDOPSIS TRITHORAX-RELATED PROTEIN 5 and 6 (ATXR5/6) (Jacob et al. 2014; Jiang and Berger 2017). The coupling of read–write activity in H3K27me3 inheritance also involves LIKE HETEROCHROMATIN PROTEIN 1 (LHP1), which binds H3K27me3 and interacts with the PRC2 subunit MULTICOPY SUPRESSOR OF IRA 1 (MSI1) (Derkacheva et al. 2013), and potentially also FERTILIZATION INDEPENDENT ENDOSPERM (FIE) (Ohad et al. 1999), the plant ortholog of the EED subunit that tethers PRC2 to H3K27me3 in animals (Margueron et al. 2009). This leads to a model where ATXR5/6 and multiple Polycomb group proteins cooperate with the replication fork to faithfully restore H3K27me3 domains during DNA replication, which is discussed in-depth in a recent review on the subject (Borg et al. 2021a). Self-sustaining read–write mechanisms are thus conserved in animals and plants, which serve to mediate the stable epigenetic inheritance of transcriptional states and cellular identity from cell-to-cell.

## Epigenetic silencing predates the origins of the plant kingdom

**Fig. 2:**
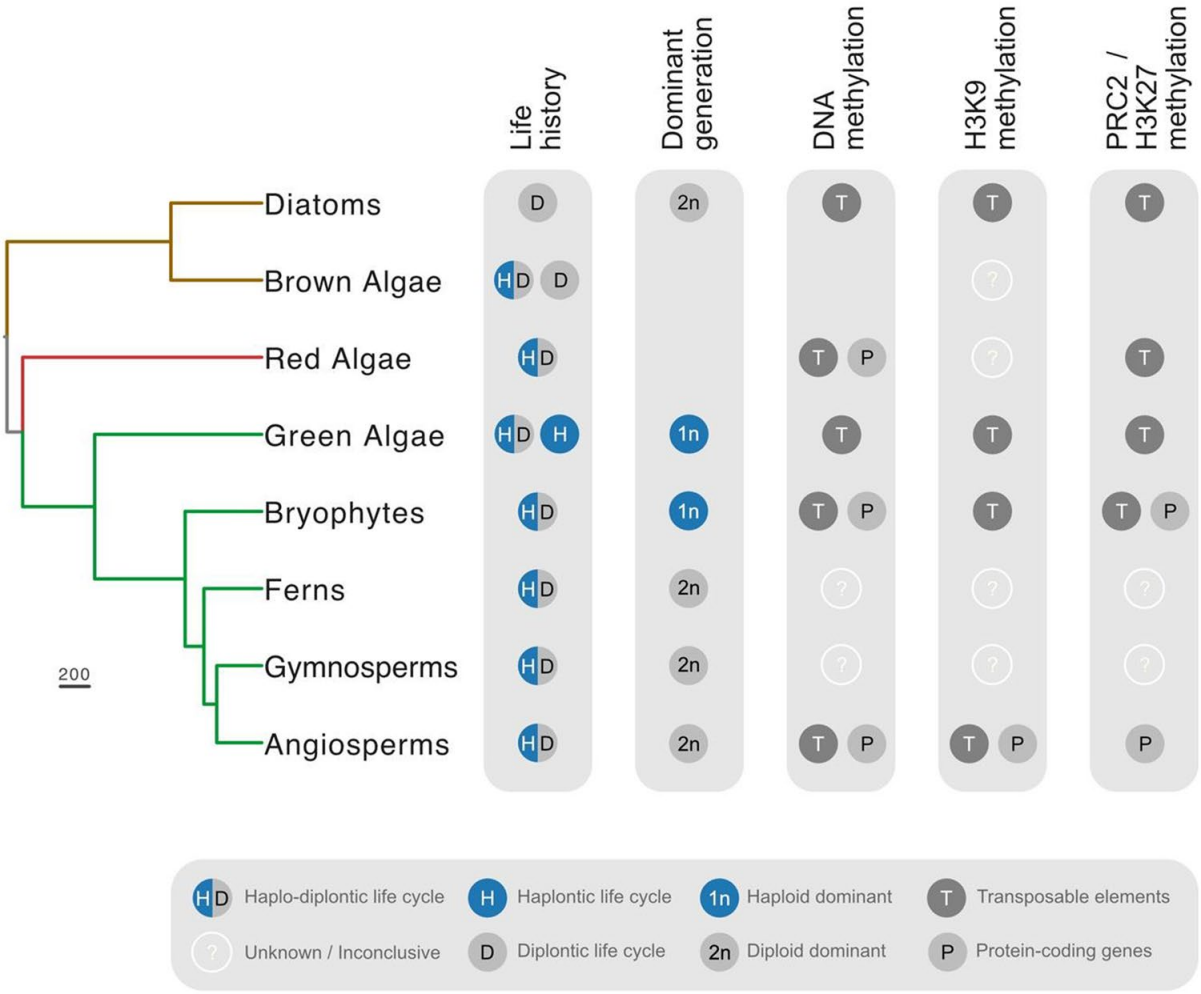
Co-evolution of life history and epigenetic silencing in plants and algae. Summary of the most predominant life history features and prevalent role played by DNA, H3K9 and H3K27 methylation in the major lineages of plants and algae. A key is provided below to denote what each circle in the diagram represents. Features absent in a particular lineage lack a circle. The summary represents the most salient features garnered in this review and is by no means exhaustive. The phylogenetic tree was constructed using TimeTree (Kumar et al. 2017) with the following representative species: *Phaeodactylum tricornutum* (Diatoms), *Ectocarpus siliculosus* (Brown algae), *Cyanidioschyzon merolae* (Red algae), *Chlamydomonas reinhardtii* (Green algae), *Marchantia polymorpha* (Bryophytes), *Ceratopteris richardii* (Ferns), *Picea abies* (Gymnosperms) and *Arabidopsis thaliana* (Angiosperms)

Our discussion will begin at the phylogenetic base of the Archaeplastida (or plant kingdom), the origins of which are traced to a primary photosynthetic endosymbiosis between a cyanobacterium and a eukaryotic host ~ 900 million years ago (Shih and Matzke 2013). The plant kingdom is represented by modern-day glaucophyte algae, red algae (or rhodophytes) and the Viridiplantae (green algae plus land plants) (Fig. 2). Algae is thus an encompassing term for a large group of diverse photosynthetic organisms from multiple eukaryotic clades, including stramenopiles like diatoms and brown algae. The concept of an alternation of generations can be misleading when applied to algae as, unlike land plants, there is often no fixed or regular alternation between two phases (John 1994). Interestingly, the term ‘alternation of generations’ arose from translation of the German term *Generationwechsel—*or *change of generation—*which is more appropriate to describe the non-obligate transitions observed in algae (John 1994). The term ‘life history’ has thus been proposed instead of ‘life cycle’ to more accurately reflect these complex changes in algal morphology and ploidy (John 1994).

Glaucophyte algae reproduce asexually through the formation of spores, with no sexual mode of reproduction reported thus far (Lopez et al. 2015). Red algae (or rhodophytes) form the largest group of mostly marine algae, some of which have high commercial value in agronomical and pharmaceutical industries. The life histories of red algae are some of the most complex known in living organisms, which is often haplo-diplontic but distinct in that triphasic life forms are expressed (Fig. 1) (Searles 1980). How this complex life history is controlled in red algae is unclear but a couple of studies have revealed that they harbor a functioning epigenetic machinery. For example, the red seaweed *Gracilariopsis chorda* is reported to have around 8% of its genome methylated (Lee et al. 2018). Much of this methylation is heavily biased to non-CG dinucleotides and is distributed equally between TEs, genes and promoters. Methylated TEs are also associated with small RNA, suggesting that RdDM might facilitate TE silencing in a manner similar to plants. The presence of H3K9 methylation and its potential synergy with DNA methylation in red algae is unknown (Fig. 2). Interestingly, at least half of all repetitive elements in the genome of the unicellular red alga *Cyanidioschyzon merolae* are marked with H3K27me3 (Mikulski et al. 2017). A small number (4%) of protein-coding genes are also marked with H3K27me3, which like H3K27me3-marked TEs, have low-to-undetectable levels of gene expression. Thus, in addition to small RNA-associated DNA methylation, H3K27me3 in red algae predominantly marks transcriptionally silent repetitive elements (Fig. 2).

Green algae are classified into two main groups—the Chlorophyte and Charophyte algae (Stewart and Mattox 1975). *Chlamydomonas* is a unicellular Chlorophyte and represents the most studied algal model organism. Aside from biotechnological applications, the phylogenetic position of *Chlamydomonas* makes it an ideal model organism to address the evolution of multicellularity in the plant kingdom. Compared to its green multicellular relatives, *Chlamydomonas* has a relatively simple haplontic life cycle with no distinct sporophytic generation (Fig. 1). The diploid zygote could thus be regarded as a sporophyte-like generation in *Chlamydomonas* and related unicellular algae, which undergoes meiosis to regenerate more haploid individuals, resulting in an alternation between haploid and diploid unicellular generations (Fig. 2). Despite this relatively simple life cycle, *Chlamydomonas* expresses a significant number of gamete-specific and zygote-specific genes, suggesting that silencing mechanisms exist to developmentally regulate these genes during its life cycle (Lopez et al. 2015).

DNA methyltransferase enzymes have ancient origins in green algae, including DNMT1 orthologs (Pei et al. 2019), while DNA methylation is detectable albeit at low (~ 1%) levels in *Chlamydomonas* and the related chlorophyte *Volvox carteri* (Babinger et al. 2007; Lopez et al. 2015). DNA methylation is confined to repeats in *Chlamydomonas* and remains stable across its life cycle (Lopez et al. 2015) but is altered during environmental adaptation (Kronholm et al. 2017), while it is linked with transgene silencing in *Volvox* (Babinger et al. 2001). Epigenetic silencing of exogenous transgenes is also reported in *Chlamydomonas*, which has been used to isolate causal silencing pathways (Jeong et al. 2002; Neupert et al. 2020). One such screen identified SET3p, a protein homologous to the *Arabidopsis* SUVH family that functions in vitro as a specific H3K9 mono-methyltransferase (Casas-Mollano et al. 2007). Knock-down expression of SET3p globally reduces H3K9me1 levels, including at the transgenic tandemly repeated arrays that are transcriptionally re-activated in its absence. The impact of SET3p knock-down on *Chlamydomonas* development was not reported, nor is it known whether H3K9me1 silences loci beyond repetitive elements. H3K9me3 is also detectable in *Chlamydomonas* but its deposition profile and catalysis still remain unknown (Casas-Mollano et al. 2007). Thus, H3K9 methylation was present in the ancestral lineage that gave rise to plants (Fig. 2), which was adapted during plant evolution to employ H3K9me2 as the dominant silencing modification of constitutive heterochromatin.

Phylogenetic identification suggest that PRC2 components were present in the common ancestor of red and green algae (Shaver et al. 2010; Huang et al. 2017b, 2019). While H3K27me3 is not detected in *Chlamydomonas* (Shaver et al. 2010; Mikulski et al. 2017; Khan et al. 2018), H3K27me1 is substantial and is catalyzed by a SET domain H3K27 methyltransferase homologous to E(z) (Shaver et al. 2010). These H3K27me1 marks co-occur on the same histone H3 tails that carry H3K4me1 (Khan et al. 2018), the latter of which appears to play an unusual transcriptional silencing role in *Chlamydomonas* (van Dijk et al. 2005). Knock-out of *Chlamydomonas* E(z) causes a global enrichment of active H3K4me3 marks and derepresses transgenes and transposons (Shaver et al. 2010), which interestingly phenocopies the loss of H3K4me1 (van Dijk et al. 2005). The tail of *Chlamydomonas* histone H3 contains an S28T mutation and deletion of residue A29 (Shaver et al. 2010), which might explain the altered mono-rather than tri-methylation at K27. This is reminiscent of H3K27me1 in *Arabidopsis*, which also silences TEs and is selectively deposited on histone H3.1 due to an A31T mutation (Jacob et al. 2009, 2014). Thus, a canonical role of PRC2-mediated silencing through deposition of H3K27me3 appears to be absent in *Chlamydomonas*, but is represented by a derived or perhaps ancestral system of transcriptional silencing that involves co-operation between H3K4me1 and H3K27me1.

## DNA methylation is essential for bryophyte development

The transition from water to land was a major event in plant evolution that profoundly impacted terrestrial landscapes on earth (Dahl and Arens 2020). The origin of early land plants traces to ancestors of aquatic green algae, with the Zygnematophyceae ‘pond scum’ thought of as the most likely sister group of land plants (Zhong et al. 2015; Puttick et al. 2018). The closest descendants of this land colonizing lineage were a diverse group of non-vascular land plants called the bryophytes, which are represented by modern-day mosses, liverworts and hornworts. The bryophyte life cycle involves obligate transitions between a dominant haploid vegetative gametophyte and a relatively short-lived diploid sporophyte (Hisanaga et al. 2019). The obligate transition in their life cycle, together with their ancient phylogenetic origins, makes bryophytes a valuable model system to explore the control and evolution of life history transitions.

The two most intensely studied bryophyte model organisms are the moss *Physcomitrella patens* and the liverwort *Marchantia polymorpha* (Rensing et al. 2008; Bowman et al. 2017), both of which harbor substantial levels of CG, CHG and CHH methylation in their genome (Schmid et al. 2018; Domb et al. 2021). While the DNA methylation machinery is largely conserved with angiosperms, CHH methylation is instead catalyzed by two DNMT3-type methyltransferases homologous to mammalian de novo methyltransferases (Cao et al. 2000; Yaari et al. 2019). The DNMT3 family became extinct in angiosperms after likely giving rise to the DRM family through rearrangement of the DNMT3 methyltransferase domain (Cao et al. 2000). DNA methylation accumulates heavily on TEs and major satellite repeats in bryophytes but, unlike seed plants, appears to be excluded from ribosomal DNA (Matyášek et al. 2019). The silencing role of DNA methylation is conserved in *Physcomitrella*, with non-CG methylation in particular playing an important role in the transcriptional repression of not only TEs but also several protein-coding genes (Fig. 2) (Domb et al. 2021).

CG methylation is maintained by orthologs of MET1, which are essential for normal gametophyte development and sexual reproduction in *Marchantia* (Ikeda et al. 2018). In contrast, *met1* gametophytes develop normally in *Physcomitrella* but fail to undergo sporophyte formation, although it is unclear whether this is caused by defects in gametogenesis, fertilization or sporophyte development (Yaari et al. 2015). However, severe gametophytic defects in *Physcomitrella* are observed in the presence of methyltransferase inhibitors (Malik et al. 2012) and in mutants of the single CMT3-type CHG methyltransferase, which incidentally also fails to form a sporophyte (Noy-Malka et al. 2014). Loss of CHH methylation in *DNMT3* mutants, on the other hand, appears to have little impact on *Physcomitrella* development since these mutants are completely viable and develop normally (Yaari et al. 2019). Interestingly, DNA methylation undergoes extensive reprogramming across the life cycle in *Marchantia* (Schmid et al. 2018). This involves substantial gains in CHH methylation at TEs and repeats during sporophyte development, as well as enriched CG and CHG methylation on gene bodies and their flanking regions within sexual organs and sperm (Schmid et al. 2018). *Marchantia* thus undergoes at least two waves of DNA methylation reprogramming during its life cycle, which occurs at both TEs and protein-coding genes. A detailed examination of the loci targeted during these reprogramming cycles, together with further profiling of mutant methylomes, might offer clearer insight into how DNA methylation impacts the transition between haploid and diploid life.

What is particularly striking is the dramatic hyper-methylation observed in *Marchantia* sperm (Schmid et al. 2018)*,* where at least half of all genes accumulate 4-methylcytosine (4mC) at CG dinucleotides (Walker et al. 2021), a form of methylation previously thought to be restricted only to prokaryotes (Ye et al. 2017; O’Brown et al. 2019). 4mC was shown to be catalyzed by two novel sperm-specific DNA methyltransferases, MpDN4MT1a/b, which are essential for sperm transcription and viability (Walker et al. 2021). How prevalent 4mC is across the *Marchantia* life cycle and other eukaryotes is unclear but its discovery raises exciting questions, not least how this DNA modification might influence genomic activity differently from 5mC. Like animals, *Marchantia* produce motile sperm and densely package sperm chromatin with protamines (Reynolds and Wolfe 1978; Borg and Berger 2015), suggesting that hyper-methylation of the paternal genome might contribute to chromatin condensation and/or influences events during early embryogenesis.

## PRC2 represses the sporophyte transition in bryophytes

As we have discussed, PRC2 is present in unicellular red and green algae, such that it was likely inherited by bryophytes during evolution of the green lineage (Fig. 2) (Huang et al. 2017b; Schubert 2019). Deletion or knock-down of E(z) orthologs causes precocious formation of sporophyte-like bodies in *Physcomitrella* gametophytes (Okano et al. 2009; Pereman et al. 2016) and gametophyte lethality in *Marchantia*, respectively (Flores-Sandoval et al. 2016). Similarly, loss of the FIE ortholog, which probably tethers PRC2 to H3K27me3 as in animals (Margueron et al. 2009), negatively impacts gametophyte development and causes the formation of sporophyte-like structures in the absence of fertilization (Mosquna et al. 2009). Other ‘readers’ of H3K27me3 appear to have arisen multiple times during evolution (Schubert 2019), including the chromodomain protein LHP1 and two bromo-adjacent homology (BAH)–plant homeodomain (PHD) proteins in *Arabidopsis* (Turck et al. 2007; Zhang et al. 2007; Li et al. 2018; Yang et al. 2018). These H3K27me3 readers appear to have ancient origins in land plants (Berke and Snel 2015; Huang et al. 2019) but also in filamentous fungi like *Neurospora crassa* (Wiles et al. 2020). A loss-of-function *lhp1* mutant in *Physcomitrella* reportedly displays pleiotropic defects in the gametophyte, although latter stages of development were not reported in this study (Dangwal et al. 2014).

Among H3K27me3-silenced genes in bryophytes are the evolutionary conserved BELL and KNOX TALE-homeodomain transcription factors (TF), which interact to activate diploid gene expression and zygote formation in *Chlamydomonas* and *Marchantia* (Lee et al. 2008; Widiez et al. 2014; Horst and Reski 2016; Horst et al. 2016; Dierschke et al. 2020). Interestingly, ectopic overexpression of BELL1 in *Physcomitrella* induces embryo and sporophyte formation in the absence of fertilization (Horst et al. 2016). This likely explains the sporophyte-like features of PRC2 mutant gametophytes since BELL1 has been shown to de-repressed in these mutants (Pereman et al. 2016). The ancient KNOX-BELL program was thus likely co-opted by PRC2 to control the gametophyte-to-sporophyte transition during land plant evolution (Fig. 2). In addition to PRC2-mediated repression, the *Physcomitrella* sporophytic program is reinforced by KNOX2 class TFs that repress the haploid program in the diploid plant body (Sakakibara et al. 2013). How *KNOX*, *BELL* and other H3K27me3-silenced genes are reprogrammed during the earliest phases of the sporophyte transition is not known. H3K27me3 is lost at hundreds of protein-coding genes during the transition from juvenile to mature gametophytes in *Physcomitrella* (Widiez et al. 2014), further implying the presence of mechanisms that reprogram H3K27me3 during development. In both animals and angiosperms, H3K27me3 is extensively reprogrammed during reproductive development through histone exchange and/or active demethylation mechanisms (Hajkova 2011; Zheng et al. 2016; Borg et al. 2020). It is thus of interest to explore mechanisms of H3K27me3 reprogramming in bryophytes since these might also contribute to the control of alternating generations.

## The origin of PRC2 lies in the silencing of transposons

A striking difference in the epigenetic landscape of *Physcomitrella* and *Marchantia* is the heterochromatic nature of TEs. Like angiosperms, TEs in *Physcomitrella* are largely marked by constitutive H3K9me2-marked heterochromatin, whereas almost 40% are marked exclusively with H3K27me3 in *Marchantia* (Fig. 2) (Widiez et al. 2014; Montgomery et al. 2020). It is worth noting that these chromatin profiles were generated from gametophytic tissue, so whether the composition of heterochromatin is altered in the sporophyte is unclear. Changes to heterochromatin are known to occur in the angiosperm female gametophyte, where the reduction in DNA and H3K9 methylation coincides with a redistribution of H3K27me3 to a large proportion of TEs (Weinhofer et al. 2010). Accumulation of H3K27me3 at TEs also occurs in mutants affecting constitutive heterochromatin in fungi, animals and even plants (Reddington et al. 2013; Jamieson et al. 2016; Rougée et al. 2019). We have already highlighted how transposons and repeats are marked by H3K27me3 in unicellular red algae and green algae (Fig. 2), which also extends to diatoms and ciliates (Veluchamy et al. 2015; Mikulski et al. 2017; Frapporti et al. 2019). H3K27me3 is also co-deposited with H3K9me3 at TEs in *Paramecium tetraurelia* by a PRC2-like SET domain methyltransferase (Frapporti et al. 2019). These observations suggest that H3K27me3 has an ancestral role in the silencing of transposable elements, perhaps even in common with H3K9 methylation (Henikoff and Ahmad 2020). *Marchantia* is particularly compelling since TE silencing is partitioned between either H3K27me3 or DNA-H3K9 methylation (Montgomery et al. 2020). The redundant function of both pathways went on to diverge in a division of labor during plant evolution, with H3K27me3 becoming dedicated to repressing gene expression and DNA-H3K9 methylation dominating the silencing of TEs.

## Ferns began the transition to a dominant sporophyte generation

After bryophytes colonized terrestrial habitats, they were challenged by a new group of plants called the pteridophytes, which developed a complex vascular system. Ferns and fern-like plants are modern-day descendants of the earliest vascular plants, which went on to dominate the flora on earth during the Carboniferous period (Dimichele and Phillips 2002). The life history of ferns is reversed from that of bryophytes in that the gametophyte generation is highly reduced compared to the vegetative sporophyte (Banks 1999). Haploid spores are produced by sporangia on the underside of the leafy fronds of the fern, which then germinate into tiny free-living gametophytes. These gametophytes will differentiate the gametes, which eventually fuse to form the embryo and re-initiate sporophyte development. In *Ceratopteris richardii* and many other ferns*,* the sex of gametophytes is determined by a male-inducing pheromone (Warne and Hickok 1991), which interestingly stimulates the transcription of several epigenetic modifiers, including H3K9, H3K27 and DNA methyltransferases (Atallah et al. 2018). Heritable epigenetic silencing through DNA methylation also appears to be functional in related ferns like *Adiantum capillus-veneris* (Tsuboi et al. 2012). Thus, ferns appear to have inherited an epigenetic machinery from a common ancestor with their sister bryophyte lineage, but how this might impact life history transitions remains to be seen (Fig. 2).

## Adaptive traits in gymnosperms indicate an epigenetic-based memory

The domination of ferns and fern-like plants was eventually overtaken by seed plants, which innovated hardy seeds to protect the embryonic sporophyte and aid its dispersal over long distances. Similarly, the innovation of microscopic gametophytes also assisted gene flow over long distances through adaptive mechanisms of pollen dispersal (Levin and Kerster 1974). While the female gametophyte remains buried within the tissues of the sporophyte and forms the embryo sac, the male gametophyte develops into a free-living pollen grain that delivers sperm for fertilization (Rudall 2006b; Rudall and Bateman 2007b). Thus, unlike bryophytes and ferns, seed plants no longer relied on water and flagellate sperm for reproduction, breaking one of the last remaining links with their aquatic cousins.

Gymnosperms represent a major group of seed-producing plants, most of which are large conifer trees with cones that bear spore-producing sporangia. Norway spruce (*Picea abies*) is one such common conifer species that has important ecological and economic value in European forests. Norway spruce harbors diverse histone methylation marks (Fuchs et al. 2008), undergoes dynamic changes in H3K27me3 and DNA methylation during embryonic tissue culture (Nakamura et al. 2020), exhibits soma clonal methylome variation (Ausin et al. 2016; Heer et al. 2018) and, like angiosperms, expresses a distinct population of small RNAs in pollen (Nakamura et al. 2019). Interestingly, breeders have noted adaptive traits in Norway spruce where epigenetic events early in development (i.e., post-meiotic megagametogenesis and seed maturation) can determine how a tree behaves and grows years later, thus allowing the expression of an altered phenology when grown in a non-native environment (Yakovlev et al. 2012). Strikingly, these adaptive traits have a strong parental effect, since differences in day length and temperature during female flowering has an effect on progeny performance while that during pollen formation does not (Johnsen et al. 1996, 2005). Maternal transmission of adaptive potential is consistent with that reported in angiosperms (Wibowo et al. 2016; Luo et al. 2020), which incidentally also extensively reprogram paternal epigenetic memory (Borg et al. 2020). This implies some form of differential epigenetic reprogramming in male and female gymnosperm gametophytes, which might explain this parentally biased adaptation to different climates.

## A pre-meitoic wave of reprogramming precedes the diploid-to-haploid transition in angiosperms

The angiosperms (or flowering plants) dominate the terrestrial vegetation on earth and thus represent the most successful and diverse group of land plants. As seed plants, angiosperms share many of the innovative attributes that arose in gymnosperms, with multiple other drivers contributing to their prominence (Augusto et al. 2014). The haploid transition begins once diploid spore mother cells undergo meiosis to produce the haploid male microspore and the female megaspore (Baroux and Autran 2015). Megaspore mother cells (MMCs) are specified from nucellar cells of the ovule primordium, while pollen mother cells (PMCs) develop from sporogenous cells of the anther. Strikingly, both the MMC and PMC in *Arabidopsis* are characterized by several cytological changes at the level of chromatin, including a general decondensed chromatin state, the depletion of linker histone H1, the eviction of replicative histone H3.1 and a drastic reduction in the levels of H3K27me3 (She et al. 2013; She and Baroux 2015; Hernandez-Lagana and Autran 2020). In the PMC, these changes in chromatin are accompanied by a modest RdDM-dependent increase in CHH methylation, which regulates the RNA splicing of genes required for normal progression through meiosis (Walker et al. 2018). Thus, diploid spore mother cells undergo reprogramming both at the level of histone and DNA methylation, which likely begins to establish an epigenetic landscape that distinguishes them from surrounding diploid sporophytic cells.

## Epigenetic reprogramming controls seed development and speciation in angiosperms

The angiosperm life cycle is largely shared with gymnosperms, with the important difference being that the microscopic gametophytes give rise to two gametes that participate in a double fertilization event. The female gametophytes develop into an embryo sac containing the egg and central cell, while the male gametophytes develop into pollen grains containing two sperm within a companion vegetative cell. Pollen delivery of the sperm to the embryo sac initiates seed development, where fertilization of the egg and central cell forms the embryo and endosperm, respectively. Many of the first epigenetic mutants in *Arabidopsis* were isolated based on phenotypes that negatively affected seed development. Maternal inheritance of *fertilization-independent seed* (*fis*) mutations causes the central cell to precociously undergo endosperm development in the absence of fertilization, which abort prematurely even if the egg is fertilized (Ohad et al. 1996; Chaudhury et al. 1997; Köhler and Makarevich 2006). These *fis* mutations were mapped to *MEDEA* (*MEA*) (Grossniklaus et al. 1998; Kiyosue et al. 1999), *FERTILIZATION‐INDEPENDENT ENDOSPERM* (*FIE*) (Ohad et al. 1999), *FIS2* (Luo et al. 1999) and *MULTI‐COPY SUPPRESSOR OF IRA1* (*MSI1*) (Köhler et al. 2003), all of which encode core subunits of the PRC2 complex. H3K27me3-mediated repression thus plays a key role during female gametophyte development and likely stabilizes cellular fate in the central cell and developing endosperm.

*MEA* and *FIS2* are only expressed from maternal alleles in the endosperm, while paternal alleles remain silenced throughout seed development (Köhler and Makarevich 2006). This phenomenon, called genomic imprinting, is a characteristic feature of gene expression in the endosperm (Gehring and Satyaki 2017). Several maternally and paternally expressed imprinted genes (MEGs and PEGs) have been identified in *Arabidopsis* and rice (Gehring et al. 2009; Moreno-Romero et al. 2019; Borg et al. 2020), whose monoallelic expression arises from the differential retention of H3K27me3 or DNA methylation during male and female gametogenesis (Gehring et al. 2009; Moreno-Romero et al. 2019; Borg et al. 2020). The central cell is homodiploid in most angiosperms due to the fusion of two nuclei during female gametophyte development (Slotkin et al. 2009; Schoft et al. 2009; Mérai et al. 2014; He et al. 2019). Karyogamy with haploid sperm thus results in an endosperm with double the maternal genome dosage, which is essential since increasing paternal dosage leads to seed abortion (Zhang 2016). This dosage-sensitivity is caused by the increased expression of PEGs, with at least four PEGs demonstrated to cause seed lethality in *Arabidopsis* (Kradolfer et al. 2013; Wolff et al. 2015; Huang et al. 2017a; Wang et al. 2018). This forms the basis for an endosperm-based post-zygotic hybridization barrier called the ‘triploid block,’ which is proposed to drive speciation in angiosperms (Köhler et al. 2010; Lafon-Placette et al. 2017; Tonosaki et al. 2018).

The central cell also undergoes epigenetic reprogramming of constitutive heterochromatin through the depletion of H3K9me2 and active DNA demethylation (Hsieh et al. 2009; Pillot et al. 2010; Ibarra et al. 2012; Park et al. 2016). The latter is controlled by DEMETER (DME), a DNA glycosylase that demethylates cytosines at several genes flanked by transposons and repeats, including MEGs like *MEA* (Choi et al. 2002; Gehring et al. 2006; Park et al. 2017). DNA demethylation appears to be critical for endosperm development since, like *fis* mutants, the maternal inheritance of *dme* mutations severely impacts seed viability (Choi et al. 2002; Gehring et al. 2006). Interestingly, two PEGs that induce the triploid block encode proteins involved in H3K9me2 homeostasis, namely *ADMETOS* (*ADM*) and *SUVH7* (Kradolfer et al. 2013; Wolff et al. 2015). ADM induces the triploid block through the ectopic accumulation of H3K9me2 at AT-rich TEs, which deregulates the expression of neighboring genes in the developing endosperm (Kradolfer et al. 2013). These observations suggest that the loss of H3K9me2 is involved in specifying aspects of central cell fate, which in turn facilitates the development of the triploid endosperm after fertilization.

## The loss of DNA-H3K9 methylation rewires gene regulatory networks during *Arabidopsis* pollen development

Like the central cell, the pollen vegetative cell also undergoes epigenetic reprogramming of DNA-H3K9 methylation (Schoft et al. 2009; Ibarra et al. 2012). This appears to be more extensive than in the central cell since pericentromeric heterochromatin is dramatically decondensed, resulting in highly diffuse chromatin in the vegetative cell nucleus (VN) (Borg and Berger 2015). This epigenetic reconfiguration is caused by several mechanisms, namely the depletion of the heterochromatin remodeller DECREASE IN DNA METHYLATION 1 (DDM1) and linker histone H1, the loss of H3K9me2 and the unloading of centromeric H3 (Slotkin et al. 2009; Schoft et al. 2009; Mérai et al. 2014; He et al. 2019). Heterochromatin decondensation facilitates active DNA demethylation by DME, a process that appears to be important for pollen germination and male fertility, at least in certain *Arabidopsis* ecotypes (Schoft et al. 2011; He et al. 2019). As a result, the loss of constitutive heterochromatin identity in the VN re-activates a handful of TEs that stimulate the production of epigenetically activated small RNAs (easiRNAs) (Slotkin et al. 2009; Calarco et al. 2012; Borges et al. 2018; Wang et al. 2020).

**Fig. 3:**
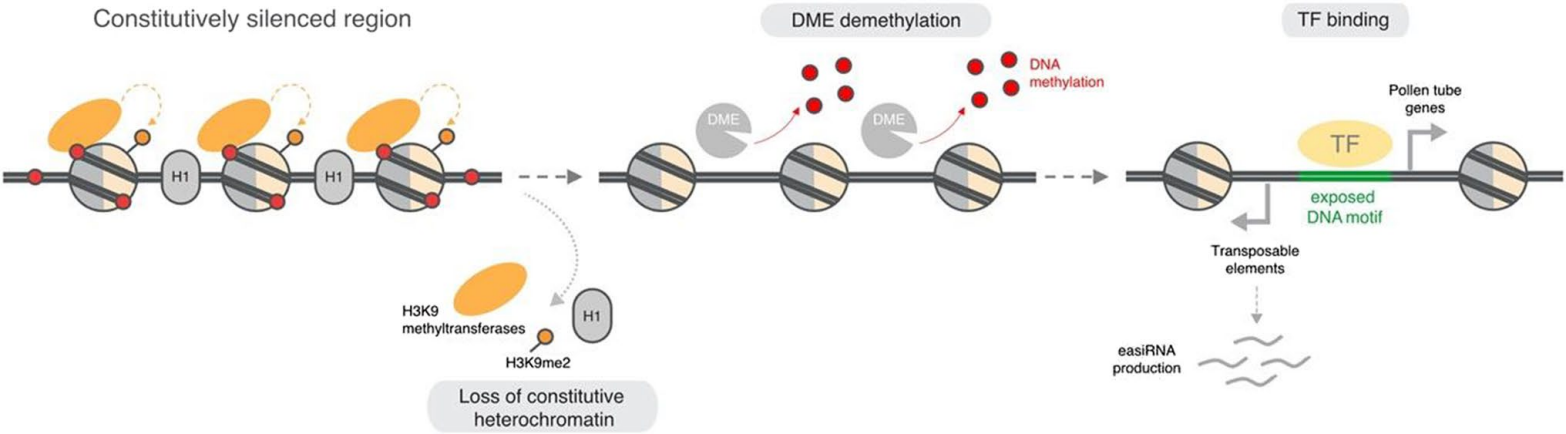
Loss of DNA-H3K9 methylation patterns the *Arabidopsis* male gametophyte. Model summarizing how the epigenetic reprogramming of DNA-H3K9 methylation in the VN activates the male gametophytic program. In the sporophyte generation, a subset of pollen-expressed genes remains silenced with constitutive heterochromatin. During pollen development, the VN undergoes extensive reprogramming to disassemble constitutive heterochromatin, which includes the loss of H3K9me2 (yellow dots) and depletion of linker histone H1 (gray ovals). The DNA glycosylase DEMETER (gray pacman) is now able to actively demethylate the high levels of DNA methylation (red dots) known to accumulate in these heterochromatic regions. This exposes *cis*-regulatory elements (green lines) in the vicinity of pollen-expressed genes normally silenced during sporophytic life, allowing methylation-sensitive TFs to bind and activate their expression. The loss of DNA-H3K9 methylation thus leads to transcriptional reprogramming and facilitates the sporophyte-to-gametophyte transition

Strikingly, a large proportion of the genomic regions targeted for demethylation by DME specifically gain chromatin accessibility in the VN (Borg et al. 2021b). This occurs within regions that not only stimulate easiRNA production but that also lie in the vicinity of VN-specific protein-coding genes, which importantly are silenced with DNA-H3K9 methylation in the sporophyte (Borg et al. 2021b). These regions are enriched for the predicted binding site of several VN-expressed TFs, many of which are unable to bind to their cognate binding sites when DNA is methylated (O’Malley et al. 2016), such that DME-mediated demethylation likely licenses their binding (Fig. 3). Consistently, DME is directly required for the expression of at least 27 of these genes, several of which have importantly been shown to play a direct role in controlling pollen tube growth (Borg et al. 2021b; Khouider et al. 2021). Thus, the reprogramming of DNA-H3K9 methylation helps rewire haploid gene expression by exposing TF binding sites normally repressed during sporophytic life (Fig. 3).

**Fig. 4:**
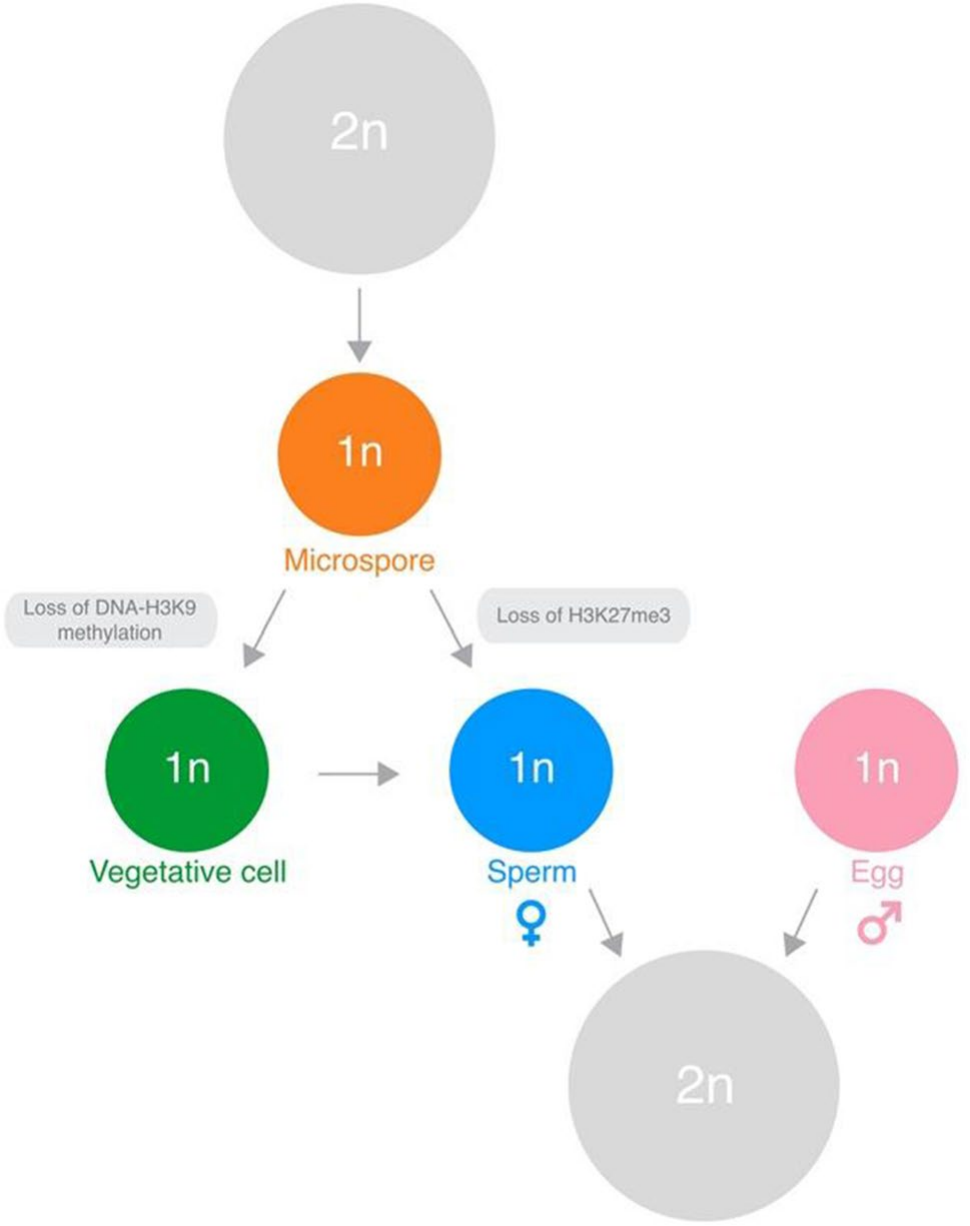
Epigenetic reprogramming in the male gametophyte guides the alternation of generations in *Arabidopsis. *The haploid epigenome undergoes extensive and differential epigenetic reprogramming during male gametophyte development. In the vegetative cell, the loss of DNA methylation and H3K9me2 relieves transcriptional silencing over pollen-expressed genes normally silenced in the diploid sporophyte to activate genes required for pollen tube growth and sperm delivery. In sperm, the loss of H3K27me3 relieves transcriptional silencing over both sperm-specific genes and master regulators of early embryogenesis. Thus, the loss of DNA-H3K9 methylation facilitates the sporophyte-to-gametophyte transition in the vegetative cell, while the loss of H3K27me3 primes the paternal genome for the gametophyte-to-sporophyte transition after fertilization

The disassembly of pericentromeric heterochromatin in the VN also impacts the regulation of ribosomal RNA in pollen. Ribosomal DNA (rDNA) genes are clustered at two nucleolar organizing regions (NORs) in *Arabidopsis* (Copenhaver and Pikaard 1996; Rabanal et al. 2017). Both NORs associate with centromeric heterochromatin and lie externally along the nucleolus in leaf nuclei, where only one NOR undergoes active transcription to produce ribosomal RNA (Mérai et al. 2014; Rabanal et al. 2017). In pollen, these NORs coalesce and become internalized in the nucleolus of the VN, which also correlates with the re-activation of rDNA variants from the silenced NOR (Mérai et al. 2014). This suggests that, in addition to pollen tube genes, rDNA loci that are normally silenced during sporophytic life become re-activated in pollen. This is likely key for increasing translational potential of the rapidly growing pollen tube. Taken together, these mechanistic insights refine our understanding of epigenetic reprogramming in the VN and suggests that its primary role is to rewire transcription and facilitate the male sporophyte-to-gametophyte transition in angiosperms (Fig. 4).

## Paternal resetting of H3K27me3 primes sporophyte development in *Arabidopsis*

In contrast to the vegetative cell, the sperm cells undergo a distinct wave of epigenetic reprogramming that drives the genome-wide loss of H3K27me3 in angiosperms (Sano and Tanaka 2010; Houben et al. 2011; Borg et al. 2020). This resetting event involves the concerted action of three mechanisms in *Arabidopsis*, namely (1) the loss of PRC2 activity and capacity to write H3K27me3 (2) active demethylation by Jumonji-C family H3K27 demethylases and (3) the sperm-specific deposition of histone H3.10, which is immune to K27 methylation (Borg et al. 2020). Almost 50% of the genes specifically expressed in sperm are silenced with H3K27me3 in the sporophyte, such that the loss of H3K27me3 facilitates their transcription during spermatogenesis. Strikingly, several H3K27me3-marked genes in the sporophyte also include master regulators of embryogenesis like *BABY BOOM* (*BBM*) and *LEAFY COTYLEDON 1* (*LEC1*) (Horstman et al. 2017; Khanday et al. 2019). In sperm, the loss of H3K27me3 as these loci coincides with increased chromatin accessibility and accumulation of active H3K4me3 modifications at promoter regions, which occurs in a pattern that predicts gene expression during earliest phases of embryogenesis (Borg et al. 2020, 2021b).

Paternal resetting of H3K27me3 thus primes the paternal genome for the sporophyte transition and has the potential to impact transcription in the early zygote. Nevertheless, these effects might be highly transient since paternal chromatin is rapidly reprogrammed in the zygote after fertilization (Ingouff et al. 2010). In contrast, paternally derived histones are passively diluted over several rounds of DNA replication in the endosperm (Ingouff et al. 2007), which might provide a longer window for histone-based imprints to act. Interestingly, unlike MEGs, many PEG loci are also transcriptionally primed with open chromatin and H3K4me3 in sperm (Borg et al. 2020, 2021b), providing correlative evidence for how paternal chromatin potentially impacts gene expression after fertilization. Mechanistically, pre-configured accessible chromatin could help elicit the binding of maternally derived TFs or perhaps already signifies a paternal contribution of pre-bound TFs at fertilization. In summary, the reprogramming of H3K27me3-silenced states in the male gametophyte generation orchestrates the rewiring of transcription to facilitate the haploid-to-diploid transition in angiosperms (Fig. 4).

## Diatoms share a chimeric epigenetic landscape with animals and plants

Stramenopiles (or heterokonts) are a monophyletic group that arose independently from Archaeplastida through a secondary endosymbiosis event involving the capture of a red alga. They include diverse unicellular algae such as diatoms and water molds (or oomycetes) but also multicellular species of brown algae. Diatoms are essential plankton components of aquatic ecosystems that are famed for their dazzling and elaborate silica cell walls. Like animals, the diatom life cycle is diplontic with the haploid phase reduced down to the gametes (Fig. 1). Methylome profiling in several different diatom species has revealed generally low levels of DNA methylation with some exceptions ranging as high as ~ 60% (Jarvis et al. 1992; Veluchamy et al. 2013; Huff and Zilberman 2014; Traller et al. 2016). Much of this methylation is restricted to TEs and a few genes, whereas intergenic regions are generally devoid of methylation. The localized enrichment of high levels of DNA methylation in the model diatom *Phaeodactylum tricornutum* suggest deep links with chromatin structure, which is dynamically altered with transcription in response to environmental changes (Veluchamy et al. 2013). DNMT1 symmetric methyltransferases are absent in diatoms (Zemach and Zilberman 2010), with CG methylation likely deposited by DNMT5 methyltransferases known to be active in other CG-methylated species lacking DNMT1 (Huff and Zilberman 2014).

*Phaeodactylum* also harbors a wide repertoire of histone modifications (Veluchamy et al. 2015). H3K27me3 covers about 14% of the *Phaeodactylum* genome, which is unusually high but coherent with its high load of H3K27me3-marked TE insertions. The fraction of H3K27me3-marked genes in *Phaeodactylum* is altered as morphotypes undergo changes in cell shape, with an E(z) knock-out abolishing these elaborate shapes altogether (Zhao et al. 2021). These findings highlight a role of PRC2 in developmental regulation and cell identity in unicellular species that may be more ancient than previously appreciated (Zhao et al. 2021). Several forms of H3K9 methylation are also found in *Phaeodactylum*, with the relevant marked loci globally anti-correlated with transcription (Veluchamy et al. 2015). Like *Paramecium* (Frapporti et al. 2019a), H3K9 methylation in *Phaeodactylum* might be under control of an E(z) homolog since no other H3K9 methyltransferases are present while H3K9 and H3K27 methylation largely co-occur. Thus, diatoms have a chimeric epigenetic landscape that shares both animal and plant features where H3K9me2, H3K27me3 and DNA methylation all participate in the repression of TE activity.

## Life history transitions in brown algae involves independent regulatory controls

Brown algae (or phaeophytes) are a large group of multicellular eukaryotes with diverse morphology ranging from tiny species like *Ectocarpus siliculosus* to 50-m-long giant kelps like *Macrocystis pyrifera*. As plants do on land, brown algae play fundamental ecological roles in supporting coastal marine ecosystems and have direct economic impact in food harvest, commercial extracts and marine fouling (Bringloe et al. 2020). Brown algae are haplo-diplontic organisms with the exception of the Fucales order which, like animals, have a diplontic life cycle (Fig. 1) (Cock et al. 2014). Developmentally, brown algae are fascinating in that they display a dazzling variety of life histories across the entire clade (Heesch et al. 2019). The transition between haploid and diploid phases is also often non-obligate in brown algae. For example, gametes in the model brown algae *Ectocarpus* can initiate the sporophyte program without fertilization, developing with or without endoreplication to become either diploid or haploid parthenosporophytes (Müller 1966; Bothwell et al. 2010). *Ectocarpus* can thus transition through a complete life cycle with the same genes and ploidy without fertilization, making it a compelling case study of the alternation of generations in brown algae.

Although they share a common ancestor with diatoms, neither DNA methylation nor its deposition machinery is detected in *Ectocarpus* (Cock et al. 2010). *Ectocarpus* has a relative simple morphology with only a few different cell types, which contrasts with the complex tissues and different cell types of giant kelps (Bringloe et al. 2020). DNA methylation has been reported in the kelp *Saccharina japonica*, albeit at very low (1.4%) levels (Fan et al. 2020). Much of this methylation appears to occur in a CHH context, anti-correlates with transcription, and targets a wide range of genomic features. The *S. japonica* genome only encodes a homolog of the tRNA methyltransferase DNMT2, leading to the supposition that these homologs methylate both RNA and DNA, although no biochemical evidence was presented to test this hypothesis. The unequivocal presence of methylated cytosines by mass spectrometry in *S. japonica* DNA is also lacking*.* Coupled with the trace amounts of DNA methylation reported and the lack of an obvious DNA methyltransferase, it is probably premature to conclude that brown algae harbor and regulate genome activity using DNA methylation.

Given how histone methylation regulates life cycle transitions in land plants, its role in brown algae is of key interest. While several histone marks are present in *Ectocarpus*, H3K27me3 is absent, while H3K9me2/3 is only detected at very low levels (Bourdareau et al. 2021). Consistently, no E(z) and PRC2 homologs have been identified in brown algae thus far, including *Ectocarpus* and *S. japonica* (Bourdareau et al. 2021). A homolog of EHMT2 (or G9a), which functions as a distinct H3K9 methyltransferase in animals, is present in *Ectocarpus* along with several known H3K9 demethylases (Maumus et al. 2011). Thus, while H3K27me3 is clearly absent, the status of H3K9 methylation remains inconclusive. *Ectocarpus* gametophytes and sporophytes are highly isomorphic, with the minimal changes in transcription reflected in the histone landscape across its life cycle (Bourdareau et al. 2021). It is worth noting that parthenosporophytes were assayed in this study, so sporophytes arising from fertilization might display a distinct profile, particularly since zygotic chromatin is known to undergo dramatic changes in plants and animals (Loppin et al. 2005; Bonnefoy et al. 2007; Ingouff et al. 2010). Thus, it remains to be resolved whether any form of epigenetic reprogramming is associated with the alternation of generations in *Ectocarpus* and whether this is common across the brown algal lineage.

## Perspectives and future endeavors

Since its first discovery by Hofmeister over 170 years ago, a mesmerizing array of life history strategies have been described that mirror the increasing complexity of plants and algae. Epigenetic silencing provides a means to reshape differentiation and is a proposed driving force in the transition to multicellular life from the ancestral unicellular state (Gombar et al. 2014). Epigenetic silencing was likely present in the common ancestor of plants, where it primarily served as a genome defense system against TEs and exogenous retroviruses but then diverged into the control of gene expression (Fig. 2). With the advent of meiosis and fertilization, the stage would have been set for epigenetic regulation to begin diverging between the haploid and diploid phases of the life cycle, ultimately giving rise to the diverse life histories observed in modern-day plants and algae.

DNA methylation is a clear case in point. In green algae, DNA methylation is restricted to gene-poor regions and is largely static across the life cycle (Lopez et al. 2015). In bryophytes, DNA methylation also marks genes, is essential for development and undergoes dynamic changes during the life cycle (Noy-Malka et al. 2014; Yaari et al. 2015; Schmid et al. 2018; Ikeda et al. 2018). In *Arabidopsis*, DNA methylation silences a subset of gametophyte-specific genes together with H3K9 methylation in the sporophyte generation, which are both reprogrammed during male gametophyte development to license activation of the haploid program (Fig. 3) (Borg et al. 2021b; Khouider et al. 2021). DNA-H3K9 methylation is reconfigured similarly in the central cell of the female gametophyte (Pillot et al. 2010; Ibarra et al. 2012; Park et al. 2016), suggesting that this mechanism probably plays a general role in the sporophyte-to-gametophyte transition in angiosperms. DNA-H3K9 methylation is normally associated with TE silencing in flowering plants (Feng and Michaels 2015), but its role in the gametophyte highlights how it also represses developmental programs that govern lineage specification. TE insertions have rewired transcriptional networks during evolution in other eukaryotic lineages (Rebollo et al. 2012), and it is interesting that many male gametophyte-specific genes activated upon the loss of DNA-H3K9 methylation are associated with TEs (Borg et al. 2021b). TE activity could have thus altered the gametophytic program in ancestral land plants, providing fertile ground for regulatory mechanisms to evolve and control haploid–diploid transitions.

Unlike H3K9 methylation, PRC2-mediated silencing through H3K27me3 is largely dedicated to regulating growth and development in complex eukaryotes (Margueron and Reinberg 2011). However, as we have discussed, the ancestral role of PRC2 was also a genome defense system that evolved toward developmental regulation and cell identity (Fig. 2). PRC2 still functions today to repress TE activity in unicellular red and green algae (Mikulski et al. 2017) but also bryophytes (Montgomery et al. 2020). At some point in the evolution of early land plants, PRC2 began to silence gene expression, which included the BELL/KNOX system that regulates the diploid program in green algae and bryophytes (Lee et al. 2008; Widiez et al. 2014; Horst and Reski 2016; Horst et al. 2016; Dierschke et al. 2020). This scenario would have led to repression of the sporophyte program and extension of the gametophytic phase, which is still evident in modern-day bryophytes. In *Arabidopsis*, the loss of H3K27me3 in sperm might have a similar role, since this relieves silencing over several master regulators that initiate the sporophyte program in angiosperms (Horstman et al. 2017; Borg et al. 2020). Thus, studies of chromatin reprogramming during male gametophyte development suggest a model where programmed loss of H3K27me3 and DNA-H3K9 methylation act independently to facilitate life cycle transitions in *Arabidopsis* (Fig. 4). Further investigation of chromatin reprogramming in the female gametophyte and other flowering plant species will help determine how general the mechanisms operating in *Arabidopsis* pollen are for the gametophyte transition in angiosperms.

The independent evolution of the alternation of generations in the Stramenopile lineage raises questions about what is common or different about its molecular control. Brown algae display a complex array of life history strategies (Heesch et al. 2019), but the clear absence of PRC2 silencing already suggests that aspects of the sporophyte transition in plants cannot be generalized (Fig. 2). Moreover, the fact that PRC2 pathways are functional and essential for cell identity in diatoms but were lost in brown algae is further compelling and suggests that novel regulatory mechanisms could have emerged during Stramenopile evolution. Despite the differences in epigenetic control, two TALE-homeodomain TFs are known to promote sporophyte formation in *Ectocarpus* (Coelho et al. 2011a; Arun et al. 2019). This mode of regulation bears a striking and remarkable similarity with the BELL/KNOX system in the green lineage. The evolution and regulation of the life history transitions is thus clearly complex and likely involves both common and lineage-specific mechanisms. Future endeavors must expand across and beyond the green lineage to provide both a molecular and evolutionary understanding of the alternation of generations, which is what ultimately makes the developmental biology of plants and algae so fascinating.
